# The Odontographic Society of Pennsylvania, and Dr. Norman Kingsley’s Obturator

**Published:** 1865-01

**Authors:** 


The Odontographic Society of Pennsylvania, and Dr. Norman Kingsley's Obturator.—Our readers will remember our directing attention during the past summer to an artificial soft palate, invented by Dr. Kingsley, of New York, and by him generously given to the profession. This appliance has since that time been very thoroughly tested, and seems destined to occupy an important place among the numberless apparatus employed by the surgeon for the relief of afflicted humanity.

In consideration of the proper professional feeling displayed by Dr. Kingsley, in the disposition of his invention, and in the pride, as well, we suppose, of the connection of the invention with the speciality of dentistry, the Odontographic Society of this city has, through its Chairman, Dr. J. H. McQuillen, presented to Dr. Kingsley, a beautiful and valuable gold medal.

One side of this medal bears the fac simile of the seal of the Association, viz: having around the margin "Odontographic Society of Pennsylvania Instituted 1863," and in the centre the Etruscan Lamp with a pen and excavator crossed, with the motto "Labor by day, study by night," in a scroll underneath; on the reverse is inscribed "Presented to Dr. Norman W. Kingsley."

In presenting the medal, Dr. McQuillen, accompanied it with some appropriate remarks, referring with just pride to the fact that, beside this invention, which he looked upon as a boon to the world; to dentistry the world is also indebted for the greatest discovery ever given it, namely, ether as an anæsthetic. The speaker congratulated himself and his fellow members upon the rapid strides being made by the dental profession in the way of scientific acquisition. Referring to the presentation of this medal, Dr. McQuillen remarked:

"There are some who not only oppose the presentation of testimonials to those who have rendered valuable services to the world, but also act and talk as if such recognition of another's claims to consideration was so much taken from themselves. Such sentiments, however, are not entertained by the members of this Society, and I do not believe that any organization in presenting a medal to a gentleman who, by his efforts, proves that he merits it, belittles itself in the estimation of properly constituted minds; for it is but making a *just* recognition of the labor which has been done, and is, in addition, an encouragement to others engaged in efforts tending to increase the comfort and happiness, or relieve the sufferings of humanity. It is not sufficient to erect monuments to the memory of those who served us, when their remains are mouldering beneath the sod, but they should receive that encouragement and support from their fellows, to which they are entitled, while they live. The prompt recognition and endorsement of a new and decided improvement is not only justifiable on such grounds, but also from the fact that such action on the part of an organization whose avowed object is to promote and encourage, among dental practitioners, a disposition for investigation and improvement in every direction which relates to the principles or practice of the profession, tends to make the improvement more widely known, and therefore more speedily and generally adopted and used. Considerations such as these prompted the motion, offered at a previous meeting, to present this medal; and I am glad, gentlemen, that to-night you have had an opportunity of seeing the *practical* value of this ingenious appliance, which some of the first surgeons of Philadelphia said to me to-day that they regarded as 'one of the greatest improvements of the age.'"

On receiving the medal Dr. Kingsley expressed himself as feeling very happy that it had fallen to his lot to be the inventor of an appliance which the members, both of his own and the medical profession, seemed disposed to think so kindly of.—*Med. & Surg. Reporter.*

